# Feasibility of high-intensity training in asthma

**DOI:** 10.1080/20018525.2018.1468714

**Published:** 2018-05-11

**Authors:** L. L. Toennesen, E. D. Soerensen, M. Hostrup, C. Porsbjerg, J. Bangsbo, V. Backer

**Affiliations:** a Department of Respiratory Medicine, Respiratory Research Unit, Bispebjerg Hospital, Copenhagen, Denmark; b Section of Integrative Physiology, Department of Nutrition, Exercise and Sports, University of Copenhagen, Copenhagen, Denmark; c IOC Research Center Copenhagen, Center for Injury Prevention and Protection of Athlete Health, Copenhagen, Denmark

**Keywords:** Asthma, high-intensity interval training, exercise, asthma control

## Abstract

**Background:** High-intensity interval training is an effective and popular training regime but its feasibility in untrained adults with asthma is insufficiently described.

**Objective:** The randomized controlled trial ‘EFFORT Asthma’ explored the effects of behavioural interventions including high-intensity interval training on clinical outcomes in nonobese sedentary adults with asthma. In this article we present a sub analysis of data aiming to evaluate if patients’ pre-intervention levels of asthma control, FEV1, airway inflammation and airway hyperresponsiveness (AHR) predicted their training response to the high-intensity interval training program, measured as increase in maximal oxygen consumption (VO_2_max).

**Design:** We used data from the EFFORT Asthma Study. Of the 36 patients randomized to the 8-week exercise intervention consisting of high-intensity training three times per week, 29 patients (45% females) completed the study and were included in this data analysis. Pre-intervention assessment included the asthma control questionnaire (ACQ), spirometry, fractional exhaled nitric oxide (FeNO) and AHR to mannitol. VO_2_ max was measured during an incremental cycle test.

**Results**: The majority of included patients had partly or uncontrolled asthma reflected by a mean (*SD*) ACQ at 1.7 (0.6). Median (IQR) FeNO was 28.5 (23.8) ppb and 75% had a positive mannitol test indicating AHR.

The association between patients’ training response measured as increase in VO_2_max and pre-intervention ACQ scores was not statistically significant (*p* = 0.49). Likewise, the association between patients’ increase in VO_2_max and FeNO as well as AHR was not statistically significant (*p* = 0.80 and *p* = 0.58).

**Conclusions**: Included asthma patients could adhere to the high-intensity interval protocol and improve their VO_2_max regardless of pre-intervention levels of asthma control, airway inflammation and AHR.

## Introduction

Engaging in physical activity can be a challenge for patients with asthma, since many experience exercise-induced asthma symptoms [1]. This may cause them to avoid physical activity, leading to deconditioning and poor cardiorespiratory fitness [2,3].

High-intensity interval training is today being practiced within a number of rehabilitation programs especially in programs for patients with cardiovascular disease [4,5], because of its superior efficacy to improve cardiorespiratory fitness and endurance performance when compared to low- to moderate-intensity training [4,6]. Our current knowledge about the feasibility of exercise interventions in asthma is limited by the fact that the majority of training studies have been based on low- and moderate-intensity training.

We recently performed the EFFORT Asthma study, an 8-week RCT exploring the effects of behavioural interventions including high-intensity interval training on clinical outcomes in nonobese sedentary adults with asthma [7]. Here we demonstrated that high-intensity training was safe and improved VO_2_max significantly in the overall group of patients randomized to follow the 8-week training program. In the present article, we aim to explore if patients’ pre-intervention levels of asthma control, FEV1, airway inflammation and airway hyperresponsiveness (AHR) predicted their training response following the high-intensity interval training program, measured as increase in maximal oxygen consumption (VO_2_max).

## Methods

The methodology has been described in detail previously [7].

## Design

The study is a sub-analysis of data from the EFFORT Asthma study [7], (ClinicalTrials.gov NCT02355964). Given the present study’s focus is an in-depth analysis of the feasibility of the high-intensity interval training, the current study only includes data from patients who were randomized to the *exercise group* (*n* = 29).

Each patient gave written informed consent and the study was approved by the local Ethics Committee of Copenhagen, Denmark (H-4-2013-116)).

## Patients

Patients were recruited consecutively through newspaper advertisements and through the outpatient asthma clinic at Bispebjerg Hospital, Copenhagen, Denmark. The inclusion criteria were as follows: (i) 18–65 years of age, (ii) a diagnosis of asthma confirmed through an interview with a study physician and a positive diagnostic test demonstrating variable airflow obstruction: either a positive mannitol test, methacholine test or reversibility test, (iii) Asthma control questionnaire score (ACQ score) ≥ 1.0, (iv) body mass index (BMI) >20 and <30 kg/m^2^, (v) untrained (less than 1 h of physical exercise per week), and (vi) on a stable treatment regime (either receiving no asthma controller medicine (i.e. inhaled corticosteroid (ICS) or ICS combined with long-acting beta-2 agonist (LABA), or on a stable dose throughout the last 3 months). Patients were excluded if they had been hospitalized for asthma within the last 3 months, had a lower respiratory tract infection within the last 3 months, and if they had a medical history of other chronic lung disease, including chronic obstructive pulmonary disease or other disease that could compromise safety.

## Study flow

After patients had signed informed consent, a screening visit was performed to assess in and exclusion criteria and confirm the diagnosis of asthma with spirometry and provocation test(s). An exercise test was performed on a separate day.

## The exercise intervention

The exercise intervention consisted of 8 weeks of high-intensity interval training using the *10-20-30* training concept [6]. The training was performed on indoor spinning bikes (TopSpin, Abilica, Denmark) three times per week and was supervised by a spinning instructor. The training comprised of a 10-min warmup at a low intensity followed by two to four 5-min intervals. Each 5-min interval consisted of five consecutive 1-min intervals divided into 30, 20 and 10 s at an intensity corresponding to <30%, <60% and >90% of maximal heart rate, respectively. Intervals were separated by 2-min recovery periods and all sessions included a 10-min cool-down period consisting of biking at a low intensity. In the first 2 weeks, patients completed *two* 5-min intervals per session, *three* intervals were completed per session during the following 3 weeks, and *four* intervals per session during the remaining 3 weeks. During every training session, patients wore a heart rate monitor (Polar h7 heart rate monitor, Polar, Denmark) which enabled them to follow their own heart rate on a large screen on the back wall. The target heart rate during each 5-min interval was 90–100% of maximal heart rate (HRmax). All heart rate data from each training session was registered and stored with the POLAR team system software (Polar, Denmark).

The spinning instructors kept a record of patients’ attendance to the training sessions and a minimum of 21 training sessions during the 8-week intervention period were required.

## Safety

Patients were instructed to take 2 puffs of their regular short-acting beta-2 agonist (SABA) 10–15 min prior to the training and during the training sessions if necessary to prevent bronchoconstriction. All training sessions were carried out in a hospital setting. To ensure patients’ safety during the training session, safety guidelines were taught to all trainers. They were taught to call the hospital’s emergency assistance team in the case of a major serious event which was defined as follows: 1) if a patient suffered from asthma symptoms that did not pass after 1–2 puffs of SABA or 2) if a patient experienced dizziness or discomfort during the training session that did not pass after a short pause from training (1–2 min.). Major serious events were systematically recorded. In contrast, *minor* events such as if a patient decided to take 1–2 puffs of extra SABA during the training session or wanted a 1–2 min break from the training were not recorded.

## Assessment

All tests were performed by trained staff members and test equipment was calibrated daily according to the manufacturers’ instructions. Prior to pulmonary function tests and provocation tests, patients were asked to withhold SABA for at least 8 h, LABA for at least 12 h, ICS for at least 24 h and leukotriene receptor antagonists and antihistamines for at least 3 days.

### Asthma control

Level of asthma control was assessed with the validated 5-item version of the Juniper ACQ [8,9]. Responses are given on a 7-point scale and the overall score is the mean of the responses (0 = totally controlled, 6 = severely uncontrolled). A cut-off point at 1.5 was used to define uncontrolled asthma [8].

### Spirometry

FEV_1_ and forced vital capacity (FVC) were measured with a handheld spirometer, EasyOne (ndd Medizintechnik AG, Zürich, Switzerland) according to the standards specified by the European Respiratory Society [10]. Predicted normal values based on sex, height and age were calculated from the National Health and Nutrition Examination Survey (NHANES) reference values [11].

### Mannitol provocation test

Patients inhaled an empty capsule followed by capsules with increasing doses of mannitol (from 5 to 635 mg) until maximum doses had been reached or a 15% reduction in FEV1 had occurred. A positive test response indicating AHR was defined as a 15% fall or more at a dose ≤ 635 mg. In addition, a response-dose ratio (RDR) was calculated as the percentage fall in FEV_1_ divided by the cumulative dose of inhaled mannitol (in mg) [12].

### Methacholine provocation test

The methacholine test was performed *only* in patients who had a negative mannitol test, in order to verify the diagnosis of asthma. Tests were carried out on a separate day from the Mannitol test. Using an inhalation-triggered dosimeter (Spira Respiratory Care Centre Ltd, Hämeenlinna, Finland), subjects inhaled nebulized isotonic saline for baseline measurement followed by 1–5 incremental doses of nebulized methacholine, as described by Crapo [13]. Two minutes after each inhaled dose, a spirometry measurement was carried out. The test was stopped and considered positive when FEV_1_ decreased by 20% or more, or after accumulating a maximal dose of 8 µmol inhaled methacholine.

### Reversibility test

Reversibility testing was performed only if patients were unable to undergo bronchial provocation tests (i.e. FEV1%pred < 70%), in order to verify the diagnosis of asthma. A significant reversibility was defined as a 12% increase in FEV_1_ (and minimum 250 mL) 15 min after 4 puffs of terbutaline [14].

### Fraction of exhaled nitric oxide (FeNO) and atopy

FeNO was analysed following the ERS/ATS recommendations [15] and skin prick tests were performed according to European standards [16].

### Exercise testing

Prior to exercise testing, patients refrained from severe physical activity for at least 24 h. The cardiopulmonary exercise test was performed in accordance with the American Thoracic Society’s guidelines [17] on a bike ergometer (Monark 839E, Stockholm, Sweden). The test started at an intensity of 50 W and increased by 2 W every 6 s until exhaustion. Oxygen uptake was simultaneously recorded breath-by-breath with a gas-analyser system (Master Screen JAEGER CPX, Viasys Healthcare, Hoechberg, Germany). Before testing, all patients were instructed to inhale two puffs of their regular SABA. VO_2_ max was measured as the highest oxygen consumption in a period of 30 s divided by the total body weight. The criteria used to end the bicycle tests were as follows: Either an elevated respiratory exchange ratio (RER) ≥1.1, achievement of at least 90 percentage of age-adjusted estimate of maximal heart rate (HRmax) and/or reaching a VO2 plateau within 2 and 2.2 ml·kg(−1)·min(−1). Peak power output (PPO) was defined as the highest load (watt) reached during the exercise test. HRmax was measured during the test.

## Statistical analyses

Data was stored and analysed using the statistical software SPSS 23 (SPSS Inc. Illinois. USA). For continuous outcomes, means (standard deviations; *SDs*) were compared across groups using one-way analysis of variance, and Kruskal–Wallis one-way analysis of variance was used when the outcome was not normally distributed. In order to enable adjustment for potential confounders, linear regression models were used to assess the effects of pre-intervention ACQ, FEV_1,_ FeNO and AHR on improvements in VO_2_ max and PPO. For categorical outcomes, proportions were compared across groups using Chi^2^-tests. Log-transformed values of RDR to mannitol were used in all analyses in order to meet normal distribution. *P* ≤ 0.05 was used as a threshold for declaring statistical significance. Results are presented as means (*SD*) unless specified.

## Results

A total of 36 patients from the EFFORT Asthma study were randomized to the training group, and of these, 29 completed the study and were included in the present data analyses. Reasons for drop-out were: a) intercurrent illness not related to the intervention (*n* = 3), b) lack of time to complete the training program (*n* = 3) and c) not able to get in contact with the patient for follow-up examinations (*n* = 1).

## Baseline characteristics

Of the 29 included patients, 55% were men, 2 out of 3 were treated with ICS, mean FEV_1_ was 84 [13] %pred and mean ACQ score was 1.7 (0.6) (Table 1). A total of 28 (97%) had a mannitol test performed, and of these, 21 (75%) had a positive test. The remaining 7 patients had a positive methacholine test, and the patient who could not perform a mannitol test due to an FEV_1_%pred <70 had a positive reversibility test.

**Table 1.** Baseline characteristics. Results are no.(%) or mean (*SD*) unless otherwise specified.

Patients (completed study)	29
Men (n (%))	16 (55)
Age (years)	39.4 (12.5)
BMI (kg/m^2^)	24.9 (2.5)
Years with asthma (years)	17 (14)
Use ICS (n (%))	22 (76)
ICS dose (Budesonide equivalents at entry, ug)	645 (440)
Smoking (n (%))	
No	21 (72)
Yes	0 (0)
Former	8 (29)
ACQ	1.7 (0.6)
ACQ < 1.5 (n (%))	18 (62)
FEV_1_%pred	84 (13)
FVC %pred	93 (10)
FEV1/FVC	0.91 (0.08)
Positive AHR (n (%)) (*n* = 28)	21 (75)
FeNO (ppb) ^a^	28.5 (23.8)
Atopy (n (%))	21 (72)

ACQ = Asthma Control Questionnaire Score ICS = Inhaled corticosteroids FEV_1_%pred. = Forced expiratory volume in 1 s in percent of predicted FVC %pred. = Forced vital capacity in percent of predicted AHR = airway hyperresponsiveness FeNO = Fraction of exhaled nitric oxide (parts per billion).

**^a^** Median (interquartile range).

## Drop-outs

A higher proportion of men dropped-out during the intervention period than women (27% vs. 7%), albeit not statistically different (*p* = 0.21). There were no significant differences in age, BMI, ACQ score, use of ICS, FEV_1_%pred., FVC %pred., FeNO or AHR to mannitol between those patients who dropped out and those who completed the study (data not shown).

## Training sessions

**Figure 1.**
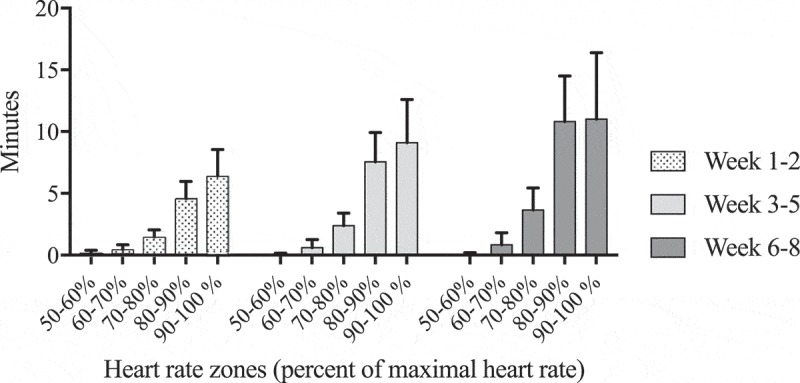
Mean time spent in each heart rate zone during one training session at week 1–2, 3–5 and 6–8.

Patients completed (mean (95% CI)) 21.9 (20.9;21.9) training sessions. No major serious events were seen. Recordings of heart rates (HR) from all attended training sessions were obtained in 26 patients (90%). Missing data was due to technical problems with the pulse sensors. The heart rates (HRs) during the training sessions relative to HRmax are shown in Figure 1. The time spent between 90 and 100% of HRmax during each of the training sessions were 6.3 (2.1) min during the first 2 weeks, 8.8 (3.6) min during the following 3 weeks, and 11.1 (5.4) min during the last 3 weeks.

Patients were able to maintain the very high intensity during the training sessions without getting asthma symptoms that could not be relieved with 1–2 puffs of SABA or a short pause (1–2 min) from training. No muscle injuries were reported beside transient muscle soreness.

## Changes in VO_2_ max, PPO and body weight

After the intervention period, mean VO_2_ max had improved from 38.4 (8.9) to 41.5 (9.5) ml/min/kg, (*p* < 0.0001), and PPO from 241 (51) to 270 (61) W, (*p* < 0.0001). A nonsignificant reduction in total body weight at 1.0 (2.2) kg was observed (Table 2).

**Table 2.** Body composition, VO_2_ max and peak power output pre-intervention and changes from pre-to post-intervention. Results are mean (*SD*).

	Patients (*n* = 29)		
	Pre-intervention	Change from pre- to post-intervention	(*p*-value)
Weight (kg)	76.4 (9.8)	**−1.0 (2.2)**	0.15
VO_2_ max (ml/min)	2921 (776)	**201 (249)**	<0.0001
VO_2_ max (ml/min/kg)	38.4 (8.9)	**3.1 (3.6)**	<0.0001
PPO (watt)	241 (51)	**28.3 (51)**	<0.0001

VO_2_ max = maximal oxygen consumption.

PPO = peak power output.

## Association between pre-intervention ACQ and changes in VO_2_ max and PPO

**Figure 2.**
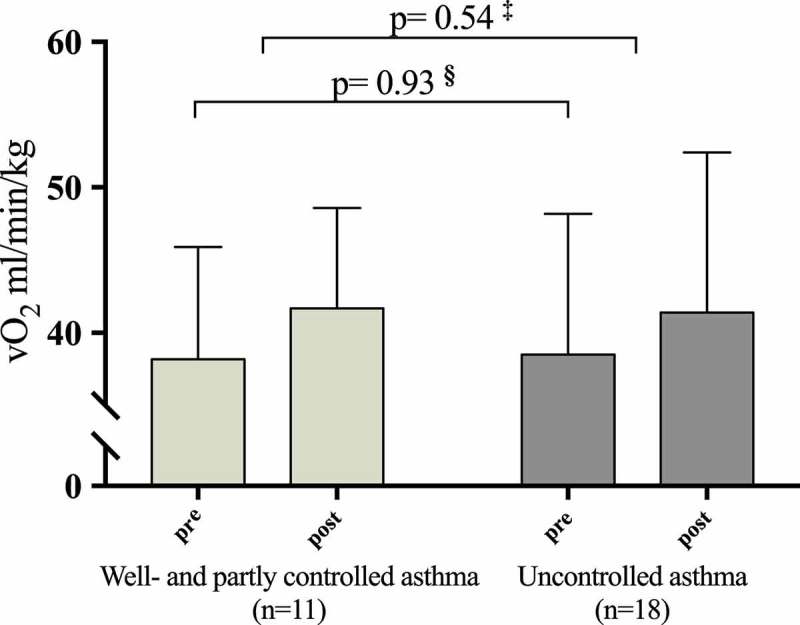
VO2 max pre- and post-intervention in patients with partly and well-controlled asthma (Asthma Control Questionnaire (ACQ) score <1.5) and uncontrolled asthma (ACQ≥1.5). **§** Pre-intervention VO2 max between patients with ACQ < and ≥ 1.5.**‡** Change in VO2 max between patients with ACQ < and ≥ 1.5.

The associations between pre-intervention ACQ scores and training response assessed as improvements in VO_2_ max and PPO were statistically nonsignificant (Table 3). Adjusting for sex and pre-intervention VO_2_ max and PPO did not change these findings. Those patients (*n* = 18) who had an ACQ ≥1.5 improved in VO_2_ max from 38.2 (7.7) to 41.7 (6.9) (ml/min/kg) which was not significantly different from the improvements obtained in the 11 patients with ACQ < 1.5, who improved ACQ from 38.5 (9.7) to 41.4 (11.0) (ml/min/kg), (*p* = 0.54) (Figure 2).

**Table 3.** Associations between clinical outcomes (pre-intervention) and improvements in VO_2_ max and peak power output following the exercise intervention.

	Improvement in VO_2_ max (ml/min/kg)						Improvement in peak power output (watt)					
Clinical outcomes (pre-intervention)	β	S.E	*P*	β adjusted^b^	S.E.	*P*	β	S.E	*P*	β adjusted^c^	S.E.	*P*
**ACQ**	−0.84	1.18	*0.49*	−0.85	1.2	*0.50*	3.7	7.8	*0.64*	4.3	8.2	*0.61*
**FEV_1_%pred.**	0.01	0.06	*0.82*	0.02	0.06	*0.81*	0.46	0.36	*0.22*	0.41	0.37	*0.28*
**AHR a** (*n* = 28)	0.84	1.5	*0.58*	0.88	5.4	*0.59*	4.2	9.8	*0.67*	5.5	10.1	*0.60*
**FeNO (ppb)**	−0.01	0.04	*0.80*	−0.01	0.04	*0.82*	0.24	0.26	*0.37*	0.30	0.29	*0.30*

FEV_1_%pred. = Forced expiratory volume in one second in percent of predicted. AHR = Airway hyperresponsiveness. FeNO (ppb) = Fractional exhaled nitric oxide (parts per billion).

^a^ Log response-dose slopes.

^b^ Adjusted for baseline VO_2_ max (ml/min/kg) and sex.

^c^ Adjusted for baseline peak power output and sex.

## Pre-intervention FEV_1_%pred and changes in VO_2_ max and PPO

The associations between pre-intervention FEV_1_%pred. and improvements in VO_2_ max and PPO were statistically nonsignificant (Table 3). Adjusting for sex and pre-intervention VO_2_ max and PPO did not change any of the findings.

## Pre-intervention FeNO and changes in VO_2_ max and PPO

The associations between pre-intervention levels of FeNO and improvements in VO_2_ max and PPO were statistically nonsignificant, and the findings remained after adjusting for sex and pre-intervention VO_2_ max and PPO (Table 3).

## Pre-intervention AHR and changes in VO_2_ max and PPO

**Figure 3.**
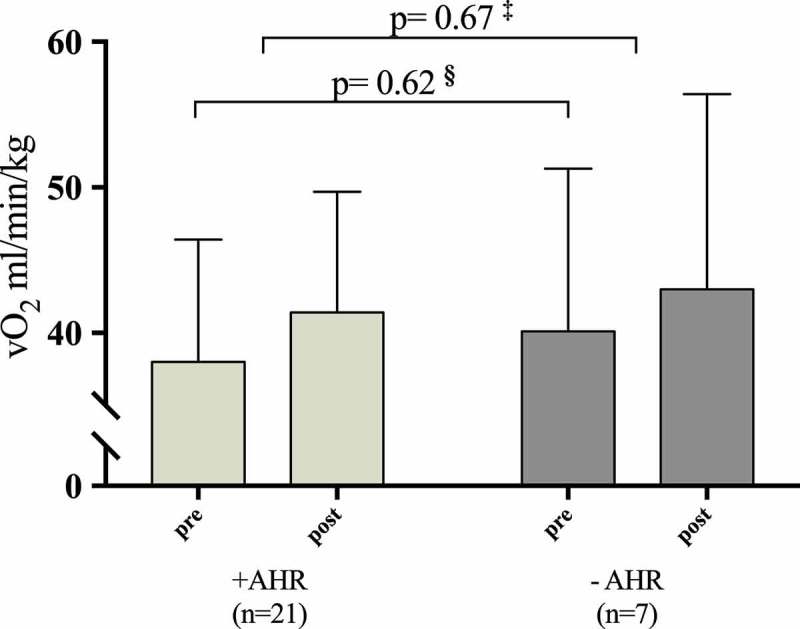
VO2 max pre- and post-intervention in patient with and without pre-intervention airway hyperresponsiveness (AHR). **§** Pre-intervention values of VO2 max between patients with a positive and a negative mannitol test.**‡** Change from pre- to post-intervention in VO2 max between patients with and without pre-intervention AHR.

The associations between pre-intervention mannitol RDR and improvements in VO_2_ max and PPO were statistically nonsignificant, and this remained after adjustment for sex and pre-intervention VO_2_ max and PPO (Table 3). Those patients (*n* = 21) who had a positive mannitol test improved in VO_2_ max from 38.0 (8.4) to 41.4 (8.3) (ml/min/kg) which was not significantly different from the improvements obtained in the 7 patients with a negative mannitol test, who improved in VO_2_ max from 40.1 (8.3) to 43.0 (13.4) (ml/min/kg), (*p* = 0.45) (Figure 3).

## Discussion

The present study demonstrates in a group of untrained asthma patients that levels of asthma control, FEV_1_, FeNO and AHR do not affect patients’ ability to engage in high-intensity training and obtain a significant training response after 8 weeks of training.

To our knowledge, this is the first study to assess this question. Only one previous study has tested high-intensity training in untrained asthma patients [18]. In this noncontrolled trial, 32 asthma patients underwent a 10-week rehabilitation program consisting of high-intensity interval training in an indoor heated swimming pool or in a gymnasium. The program included training 5 days/week during the first 2 weeks followed by 8 weeks of training three times/w *plus* an education program (physiology, medication, inhalation and breathing techniques). The training both on land and in water was found to be safe and improved exercise capacity and asthma symptoms. However, when compared to the training program in the EFFORT Asthma study, the program was rather comprehensive and it might not be feasible in every clinical setting to train in indoor pools. With the indoor spinning program we have proven the feasibility of a real-life training setup that could easily be applied inside a rehabilitation centre or in hospital training facility. The structure and progression of training through the 8-week training period was simple, and it allowed on-going enrolment of patients’ to the program, which can be an advantage in a real-life setting. Furthermore, the use of heart-rate monitors enabled the instructors to ensure patients’ adherence to the high-intensity protocol during training.

We observed a mean improvement in VO_2_ max of ~8%, which is comparable to the findings of other studies of exercise interventions in asthma. In the study by França-Pinto et al [19], patients exercised for 30 min two times/w for 3 months on a treadmill at intensities between 60 and 80% of HRmax. The average improvement in VO_2_ max was ~3.5%. Dogra et al [20] performed a non-randomized study including 12 weeks of supervised aerobic training twice weekly plus resistance training once a week, which led to an increase in VO_2_ max of ~9.5%. This indicates that though our high-intensity training program lasted just 8 weeks, it effectively improved VO_2_ max when compared to previous studies. Furthermore, when looking at findings from exercise intervention studies in healthy sedentary volunteers, the observed magnitude of VO_2_ max improvements among our included asthma patients were at a comparable level [21].

It could be anticipated that patients who experience exercise-induced asthma would be less capable of completing high-intensity training. As exercised-induced asthma is associated with AHR to mannitol, a provocation test with mannitol can be regarded as a surrogate marker of exercise-induced asthma [22]. We here report that having AHR to mannitol did not affect asthma patients’ ability to comply to high-intensity interval training and thus suggests that exercise-induced asthma should not be considered a contraindication for engaging in high-intensity interval training.

There are limitations to our study. First of all, it is not known if the included patients are a representative sample of asthma patients in the general population, and thus the magnitude of external validity of our findings. The majority of patients were enrolled after they had responded to a newspaper advertisement, and it is likely that this introduced a certain degree of selection bias. Furthermore, though included patients ranged in degree of AHR to mannitol from mild to severe and all had ACQ scores ≥1.0 indicating partly or uncontrolled asthma, patients who were on oral corticosteroids or biological treatment were not included and no patients received ICS in doses ≥1600 ug budesonide equivalents per day plus a second controller (indicating severe asthma). Our findings may therefore be most applicable to patients with mild to moderate asthma and should not be assumed to apply also in more severe disease.

In conclusion, we demonstrate here that high-intensity interval training on indoor bikes effectively improves VO_2_ max and PPO in untrained asthma patients *regardless* of their levels of asthma control, FEV_1_, FeNO and AHR. These findings suggest that clinicians should encourage asthma patients to engage in high-intensity interval training.
